# Stratified analysis of multiple management of gastric cancer: A population-based study of incidence, mortality and DALY

**DOI:** 10.1097/MD.0000000000031341

**Published:** 2022-10-28

**Authors:** Linlin Zheng, Ping Zhao, Hang Ding, Yunhui Zhou, Ningning Liu, Xinyi Zhou, Xiaohua Kong, Lin Zhou

**Affiliations:** a Department of Gastroenterology, The First Affiliated Hospital of Zhengzhou University, Zhengzhou, China; b Department of Gastroenterology, First Affiliated Hospital of Nanjing Medical University, Nanjing, China.

**Keywords:** gastric cancer, incidence, mortality, multiple management, stratified analysis

## Abstract

The aim was to illuminate the difference in incidence, mortality, and disability-adjusted life-years (DALYs) of gastric cancer (GC) between the United States of America (US) and China. The multiple management was analyzed with stratification to explore an effective survival improvement strategy. The Global Burden of Disease Study data was analyzed to assess GC morbidity, mortality and DALYs from 1990 to 2019 in the US and China. The age-period-cohort model was established to generate estimation of metrics. Verification was completed and stratified analysis of the multiple management was performed by accessing data of Surveillance, Epidemiology, and End Results database in 1992 to 2019. Continuous downtrends in GC incidence, mortality and DALYs from 1990 to 2019 and persistent uptrends in 1-, 3-year survival from 1992 to 2019 were observed in the US population. In the Chinese population, the overall trends of incidence, mortality and DALYs decreased with a fluctuating manner. The lower overall survival rates were observed in elderly, unmarried patients, distant disease and poor grade, as well as patients lacking of medical treatment (*P* < .05). In stratified analyses, single local therapy decreased and the other modalities increased over time across different stages. Moreover, combined treatment and single systemic therapy decreased, but single local and conservative therapy increased with age. The study quantified the incidence, GC-specific mortality and DALYs in the US and China and estimated stage profiles, 1- and 3-year survival in the US. The heavy burden on later-onset GC (>70) and potential increase on early-onset GC (<40) needed to be addressed. Combined modalities and single chemotherapy were becoming more widely used over time, however, their uses decreased with age because of poor physical fitness. Our findings provide new insights into management tailoring appropriately to specific subgroups contributes to the increasing survival rate.

## 1. Introduction

Gastric cancer (GC) is now the leading threat to human health globally. The case number and death rate have persistently increased from 1990 to 2017.^[1]^ Though the global incidence rate is declining, it is still the sixth leading cause of cancer incidence.^[2]^ Due to the poor prognosis,^[3]^ GC became the third most common cause of cancer-related death and disability-adjusted life-years (DALYs) for both sexes.^[2]^ Previous studies pointed that GC incidence rates were in dynamic change among subgroups,^[4]^ however, the differences of treatment modalities among subgroups were not fully elucidated. The variations in rates between subgroups with different characteristics prove the success of the public health strategies theoretically and its continuous effects on stress relief in the specific context of GC are encouraging.

The age-period-cohort models of GC in the US and China are needed since it can determine the impacts on disease incidence and mortality of different ages, periods and birth cohorts, the model can estimate the impact of age, historical events, environmental factors, and generational characteristics including risk factors and exposure to environmental factors to the early life of patients.^[5]^ The present study aimed to provide a more nuanced trend in the incidence, mortality and DALYs of GC in the US and China from 1990 to 2019, with joinpoint and age-period-cohort models based on Global Burden of Disease Study (GBD) data.

In addition, analyses based upon Surveillance, Epidemiology, and End Results databases (SEER) cancer registries 12 during 1992 to 2019 were used to validate the above trends and thoroughly illustrate multiple management by subgroups in the US population. The changes in treatment patterns categorized by risk factors over time contribute to the formulation of rational medical policies. In total, this study gives theoretical supports to alleviate the GC burden through comprehensive cancer control strategies including early diagnosis and effective treatment.

## 2. Methods

### 2.1. Study population and data processing

Data for the GC burden of the US and China and the standard population were obtained from an online source tool (http://ghdx.healthdata.org/gbd-results-tool) based on 2019 GBD study, which is an ongoing global collaboration to enable the comparable assessment of cancer burden across locations and period.

SEER*stat software (version 8.4.1) was used to retrieve cases from the SEER 12 cancer registries database, which is a public national database that covers about 13.4% of the US population. Site codes were adopted by the International Classification of Disease for Oncology-Third Edition (ICD-O-3) codes for stomach (C16.0-C16.9). The ICD-O-3 morphological codes included 8013, 8020, 8041, 8070, 8082, 8140, 8144, 8145, 8211, 8214, 8244, 8246, 8255, 8260, 8480, 8490, 8510, 8512, 8560 and 8576. Patients’ clinical characteristics (age, sex, race, marital status, treatment, and survival months), pathological (tumor primary site, stage, and grade), and vital status recode were collected from the SEER database. Age as a continuous variable was separated into 14 categories (20–24, 25–29, …, 80–84, 85+). The race was divided into White, Black, Asian, Pacific Islander and American Indian/Alaska Native, and chemotherapy and radiotherapy were separated into “No/Unknown” or “Yes.” Overall survival (OS) was defined as the time interval from the pathological diagnosis to the death or the last follow-up. Given the aggressiveness of GC, 1- and 3-year OS would be sufficient to capture enough GC-related end-points. The exclusion criteria of cases in the stratified analysis were: survival time of patients was either 0 months or unknown; and patients with absent or incomplete data regarding local therapy. The research was conducted according to the principles of the Declaration of Helsinki and the Ethics Committee of the First Affiliated Hospital of Zhengzhou University exempted the study from ethical review. Informed consent was waived for data retrieved from a public database.

### 2.2. Patient and public involvement

There was no patient privacy involved in the study, and only the information related to the patients’ diseases was retained.

### 2.3. Statistical analysis

The joinpoint models were conducted based on the incidence, mortality and DALYs in the US and China in GBD study. All rates were reported per 100,000 person-years. And the age-standardized rates were calculated based on the GBD world population standard. The age-adjusted incidence (AAI), incidence-based mortality (IBM), 1- and 3-year survival were calculated by sex using the United States Standard Population in 2000 from SEER database. Median OS was calculated by stages using Kaplan–Meier analysis. All the trends were performed joinpoint analysis and fitted with up to a maximum of 3 joinpoints. Annual percentage changes (APCs) and average APCs (AAPCs) and the corresponding 95% confidence interval (CI) were calculated to conform the trends.

The NCI’s tools (https://analysistools.cancer.gov/apc/) were used to perform the age-period-cohort analyses of incidence and mortality in the US and China. 6 equally spaced 5-year calendar periods (1990–1994, 1995–1999, …, 2015–2019) and 13 5-year age groups (20–24, 25–29, …, 80–84) were used for the analyses. The number of incident or death events by age group for a given calendar period was input as the count and the corresponding person-year at-risk individuals were input as the population. We calculated the rate ratios (RR) of incidence and mortality rates in any given calendar period (or birth cohort) versus a referent period (or birth cohort).

Continuous variables were presented as means and standard deviations, and categorical variables were presented as cases and percentages. The univariate Cox analyses were performed to determine whether the variable was a prognostic factor. We adapted t-test to compare the difference in male and female ratios at different rates.

The number of cases of stage stratification in every year over the study period was separately counted. The proportions of each treatment in different stages were calculated by year and shown by the area chart. The number of cases of different age groups was counted. The proportions of each treatment were respectively calculated by age subgroups and shown by the stacked bar chart.

All tests were used the JoinPoint Trend Analysis Software (US National Cancer Institute, Bethesda, MD; version 4.9.0.0, March 2021), SPSS software (IBM Corporation, Armonk, NY; version 26.0), and R (R Foundation for Statistical Computing, Vienna, Austria; version 4.1.1). The statistical significance level was two-sided *P* < .05.

## 3. Results

### 3.1. Age-period-cohort analyses for incidence and mortality in the US and China

**Figure 1.**
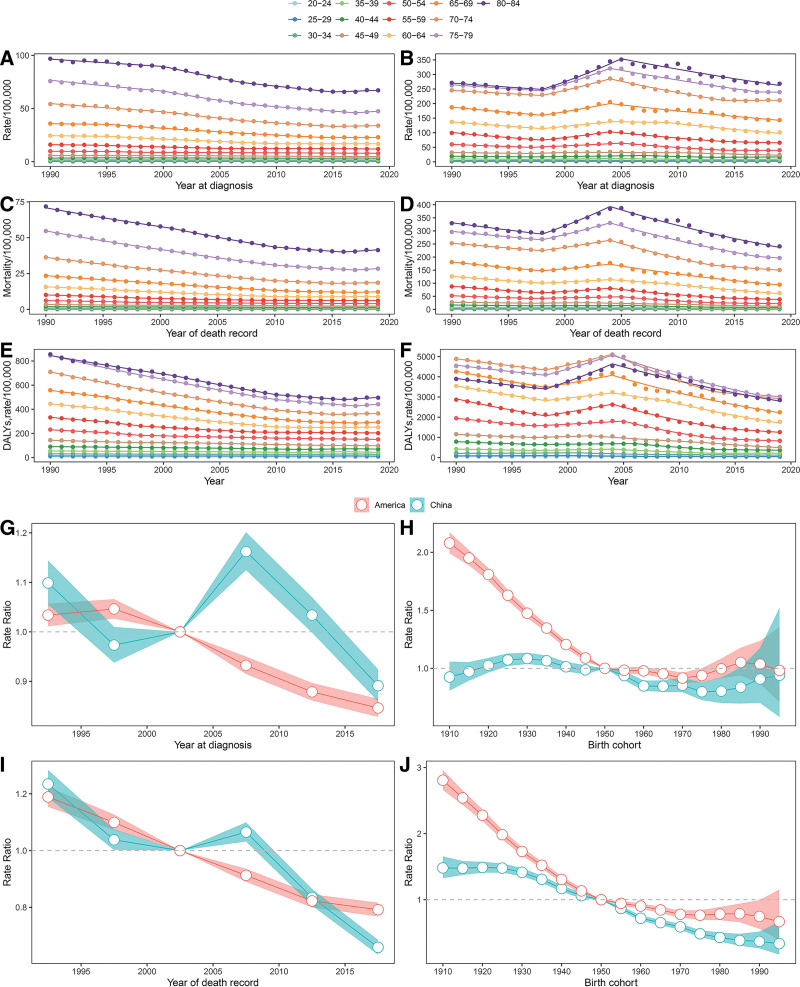
Incidence, mortality and DALY trends of GC and age-period cohort analyses, GBD study, 1990–2019. Incidence (A, B), mortality (C, D), and DALY (E, F) trends of GC in the US and China were shown in Figure 1, respectively. Points represent the actual value. Line segments of each curve in “A–F” were selected with the Joinpoint regression program. The period rate ratio (G), birth cohort rate ratio (H) of GC incidence, period rate ratio (I) and birth cohort rate ratio (J) of GC mortality were also shown in Figure 1. Color fill zones indicate 95% confidence intervals. DALY = disability adjusted life year, GC = gastric cancer; GBD = global burden of disease.

Incidence of 20 to 34 age group increased in 1990 to 2000, followed by fluctuation, and it was higher at the study endpoint (2019) than that at the beginning (1990) in both the US and Chinese population (Fig. 1A and B). For the US population, incidence of 35 to 44 went through the same process twice, first increase and then decrease, and the incidence in 2019 was slightly lower than that in 1990. Mortality of younger than 30 had a plateau during the observational period and mortality of 30 to 44 decreased as a whole with a small rise (2012–2017) (Fig. 1C). The overall incidence and mortality trends of 45 to 64 have steadily declined. The incidence of seniors (65–85) continuously decreased in 1990 to 2015, and then slightly increased, and the mortality correspondingly increased.

In Chinese population, mortality of 20 to 24 presented two plateaus, one was observed from 1990 to 1999 and the other was observed from 2013 to 2019, and a middle downturn in 1999 to 2013. Mortality of 25 to 29 went through the same process twice, first increase and then decrease. Mortality of 30 to 34 stably increased in 1990–2001 and steeply decreased in 2001–2006 and then modestly decreased in 2006–2019. For Chinese older than 35, overall incidence and mortality decreased in 1990 to 1997, and increased during 1997 to 2004 and then modestly declined until 2019 (Fig. 1B and D).

Figure 1E and F show the overall trends of DALYs for the US and Chinese population increased with age. There were some exceptions. DALYs for Chinese elder than 70 decreased with age in 1990 to 2005 and DALYs of Chinese 80 to 84 were always lower than those of 75 to 79. In horizontal comparison, the overall DALYs of Chinese older than 35 generally decreased with a rise of 1997 to 2004. DALYs of Chinese 20 to 34 increased in 1990–2000, following by fluctuation. The overall DALYs in the US population decreased over time while there was a rise of 2013 to 2017 in 25–44 age group and a rise of 2016 to 2019 in 70–84 age group.

Table 1 presents the AAPCs in the rates during the study period. It was not difficult to summarize that incidence of 70 to 79, death and DALYs of 70 to 74 had the fastest decline in the US population, and at the same time incidence of 55 to 59, death of 50 to 54 and DALYs of 50 to 59 fell fastest among Chinese groups. The incidence of Americans 20–29 and 35–44 and Chinese 80–84 had insignificant decrease while Americans 30–34 and Chinese 20–34 had significant increase.

**Table 1:** AAPCs of American and Chinese patients in different age groups during 1990–2019.

Age group	the United States of America			China		
	Incidence rates/100,000	Death rates/100,000	DALYs/100,000	Incidence rates/100,000	Death rates/100,000	DALYs/100,000
20–24	−0.1 (−0.6, 0.3)	−0.9 (−1.5, −0.3)*	−0.9 (−1.5, −0.3)*	0.5 (−0.2, 1.2)*	−1.6 (−2.1, −1.2)*	−1.6 (−2.1, −1.1)*
25–29	0.2 (−0.4, 0.7)	−0.5 (−1.4, 0.4)*	−0.5 (−1.4, 0.4)*	0.8 (−0.2, 1.8)*	−1.5 (−2.9, −0.1)*	−1.5 (−2.9, −0.1)*
30–34	0.2 (−0.1, 0.5)*	−0.5 (−0.8, −0.2)*	−0.5 (−0.8, −0.2)*	0.4 (−0.6, 1.5)*	−1.9 (−2.5, −1.3)*	−1.9 (−2.5, −1.3)*
35–39	−0.1 (−0.7, 0.4)	−0.8 (−1.1, −0.5)*	−0.8 (−1.1, −0.5)*	−0.2 (−0.5, 0.1)*	−2.3 (−2.5, −2)*	−2.2 (−2.4, −2)*
40–44	−0.1 (−0.6, 0.4)	−0.9 (−1.2, −0.6)*	−0.9 (−1.2, −0.6)*	−0.5 (−0.8, −0.2)*	−2.7 (−2.9, −2.5)*	−2.6 (−2.8, −2.4)*
45–49	−0.6 (−0.7, −0.5)*	−1.3 (−1.5, −1.2)*	−1.3 (−1.5, −1.2)*	−1.1 (−1.9, −0.4)*	−2.8 (−3.4, −2.3)*	−2.8 (−3.4, −2.3)*
50–54	−0.7 (−1, −0.4)*	−1.5 (−1.7, −1.2)*	−1.5 (−1.7, −1.2)*	−1.4 (−1.7, −1.2)*	−3 (−3.2, −2.7)*	−2.9 (−3.2, −2.7)*
55–59	−1 (−1.2, −0.8)*	−1.7 (−2, −1.4)*	−1.7 (−1.9, −1.4)*	−1.5 (−1.8, −1.2)*	−2.9 (−3.3, −2.5)*	−2.9 (−3.2, −2.5)*
60–64	−1.3 (−1.4, −1.1)*	−2 (−2.2, −1.7)*	−1.9 (−2.2, −1.7)*	−1.2 (−1.5, −0.8)*	−2.5 (−2.8, −2.2)*	−2.5 (−2.8, −2.2)*
65–69	−1.5 (−1.7, −1.4)*	−2.2 (−2.4, −2.1)*	−2.2 (−2.4, −2)*	−0.9 (−1.4, −0.5)*	−2.3 (−2.6, −1.9)*	−2.2 (−2.6, −1.9)*
70–74	−1.6 (−1.8, −1.4)*	−2.3 (−2.4, −2.1)*	−2.3 (−2.4, −2.1)*	−0.5 (−0.7, −0.3)*	−1.8 (−2, −1.6)*	−1.8 (−1.9, −1.6)*
75–79	−1.6 (−1.9, −1.4)*	−2.2 (−2.4, −2.1)*	−2.2 (−2.4, −2)*	−0.3 (−0.7, 0)*	−1.4 (−1.7, −1.1)*	−1.4 (−1.7, −1.1)*
80–84	−1.2 (−1.4, −1.1)*	−1.8 (−2, −1.7)*	−1.8 (−1.9, −1.7)*	−0.1 (−0.5, 0.3)	−1.2 (−1.5, −0.8)*	−1.2 (−1.5, −0.8)*

AAPC = average annual percent change, DALY = disability adjusted life year.

The asterisk indicated *P* < .05.

There was a notable period effect during 2000 to 2004 regarding the incidence and mortality of the two countries (Fig. 1G–I). The RRs of American incidence and mortality went up during 1990 to 1999 before the downtrend over the period of 2005 to 2019. The RRs of Chinese incidence went up during 1990–1994 and 2005–2014 before the downtrend in 2015 to 2019. The RRs of Chinese mortality went up during 1990–1994 and 2005–2009 before the downtrend in 2010 to 2019. The RRs of period effect and relevant 95% CIs were shown in Table 2.

**Table 2:** The ratio rates of period effects on incidence and mortality in China and the US.

Period	China		the United States of America	
	Incidence	Mortality	Incidence	Mortality
1990–1994	1.1 (1.06, 1.14)	1.23 (1.19, 1.28)	1.03 (1.01, 1.06)	1.19 (1.15, 1.22)
1995–1999	0.97 (0.94, 1.01)	1.04 (1, 1.07)	1.05 (1.03, 1.07)	1.1 (1.07, 1.13)
2000–2004	Reference	Reference	Reference	Reference
2005–2009	1.16 (1.12, 1.2)	1.07 (1.03, 1.1)	0.93 (0.92, 0.95)	0.91 (0.89, 0.94)
2010–2014	1.03 (1, 1.07)	0.83 (0.81, 0.86)	0.88 (0.86, 0.9)	0.82 (0.8, 0.85)
2015–2019	0.89 (0.86, 0.92)	0.66 (0.63, 0.69)	0.85 (0.83, 0.86)	0.79 (0.77, 0.82)

The cohort effect analyses of incidence and mortality were further illustrated in Figure 1H–J. In China, the RRs of cohort effect on incidence increased from 1910 to 1930 and decreased in 1931–1980 and then increased during 1981 to 1995. Meanwhile, the RRs of mortality increased in 1910–1920 and then decreased in 1921–1995 in China. The RRs of cohort effect on American incidence and mortality decreased in 1910 to 1970, following by a slight increase in 1970 to 1985 and then a decrease during 1985 to 1995. The RRs of cohort effects and relevant 95% CIs were shown in Table 3.

**Table 3:** The ratio rates of cohort effects on incidence and mortality in China and the US.

Cohort	China		the United States of America	
	Incidence	Mortality	Incidence	Mortality
1910	0.93 (0.81, 1.06)	1.48 (1.33, 1.65)	2.08 (1.99, 2.17)	2.81 (2.66, 2.96)
1915	0.97 (0.89, 1.05)	1.48 (1.38, 1.59)	1.95 (1.89, 2.02)	2.54 (2.44, 2.65)
1920	1.03 (0.96, 1.09)	1.49 (1.41, 1.57)	1.81 (1.76, 1.86)	2.28 (2.19, 2.37)
1925	1.07 (1.02, 1.13)	1.48 (1.41, 1.55)	1.63 (1.59, 1.68)	1.98 (1.91, 2.06)
1930	1.08 (1.04, 1.13)	1.42 (1.36, 1.48)	1.47 (1.44, 1.51)	1.73 (1.67, 1.79)
1935	1.07 (1.02, 1.11)	1.31 (1.26, 1.36)	1.35 (1.31, 1.38)	1.52 (1.47, 1.57)
1940	1.01 (0.97, 1.06)	1.17 (1.12, 1.22)	1.21 (1.17, 1.24)	1.31 (1.26, 1.35)
1945	0.99 (0.95, 1.03)	1.06 (1.02, 1.1)	1.09 (1.06, 1.12)	1.13 (1.09, 1.17)
1950	Reference	Reference	Reference	Reference
1955	0.94 (0.9, 0.98)	0.87 (0.83, 0.91)	0.98 (0.96, 1.01)	0.94 (0.91, 0.98)
1960	0.85 (0.81, 0.89)	0.72 (0.68, 0.76)	0.98 (0.95, 1.01)	0.9 (0.87, 0.94)
1965	0.85 (0.8, 0.9)	0.65 (0.61, 0.7)	0.95 (0.92, 0.99)	0.85 (0.8, 0.89)
1970	0.85 (0.8, 0.92)	0.59 (0.55, 0.64)	0.92 (0.87, 0.96)	0.78 (0.73, 0.84)
1975	0.8 (0.73, 0.88)	0.48 (0.44, 0.54)	0.94 (0.88, 1)	0.77 (0.7, 0.84)
1980	0.8 (0.7, 0.92)	0.43 (0.37, 0.5)	1 (0.92, 1.08)	0.78 (0.69, 0.89)
1985	0.84 (0.7, 1.01)	0.38 (0.3, 0.48)	1.05 (0.94, 1.18)	0.79 (0.66, 0.94)
1990	0.91 (0.7, 1.17)	0.37 (0.26, 0.52)	1.04 (0.87, 1.24)	0.75 (0.56, 0.99)
1995	0.94 (0.58, 1.52)	0.34 (0.17, 0.66)	0.98 (0.71, 1.36)	0.67 (0.39, 1.15)

### 3.2. Trends of incidence and incidence-based mortality in the US

**Figure 2.**
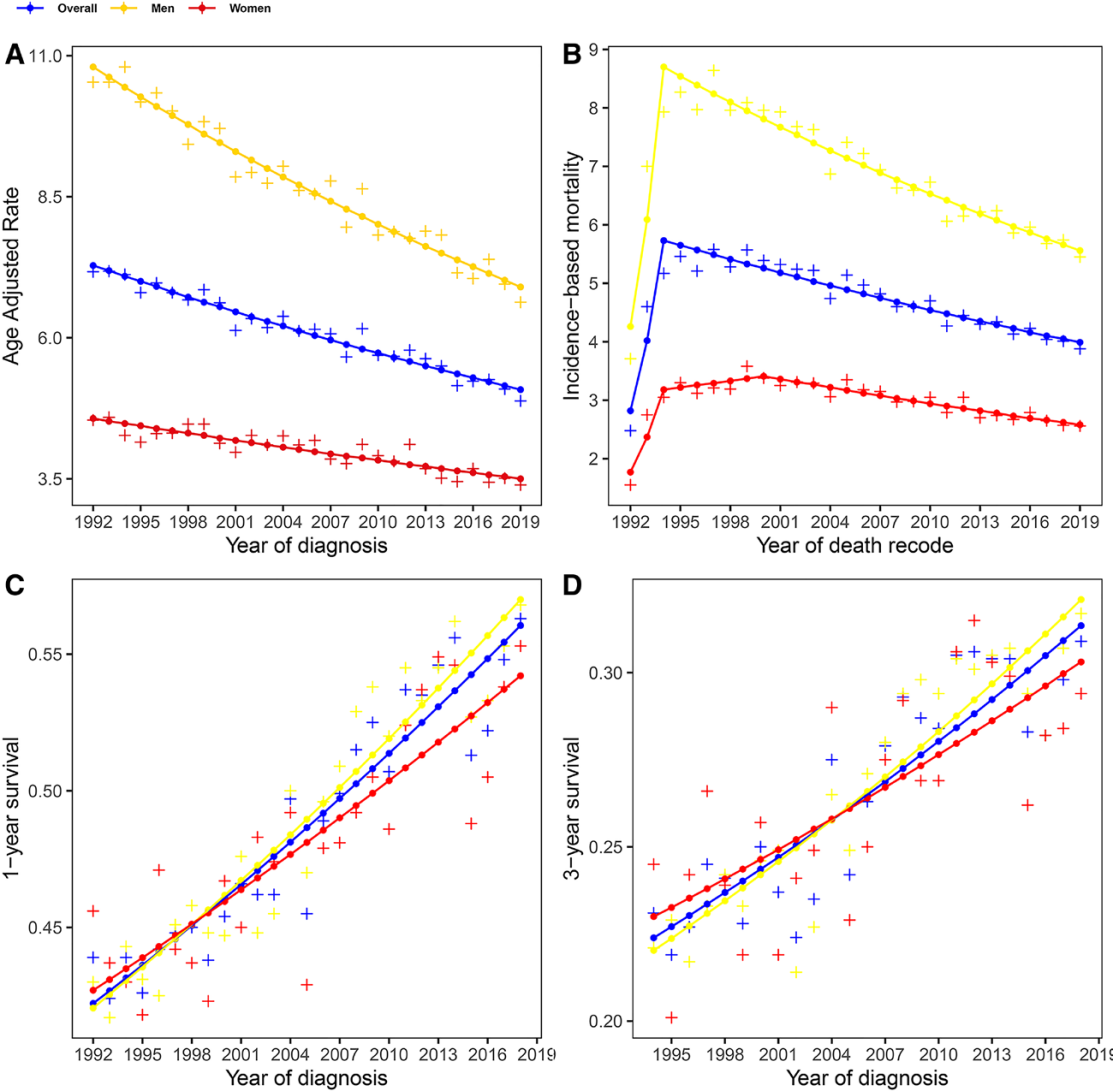
The age-adjusted incidence (A), the incidence-based mortality (B), 1-yr survival (C), and 3-yr survival (D) of gastric cancer in the Surveillance, Epidemiology, and End Results 12 program from 1992 to 2019. The scattered plus represent the observed value. The points and lines were estimated by joinpont model.

In total, 55,510 patients were enrolled in this study and 20,662 (37.2%) were female. Baseline data was shown in Table 4. With the decreasing GC incidence, the mortality presented a brief rise and then a steady decline (Fig. 2A and B). The AAI continued to decline at 1.3% per year significantly (95% CI: −1.4% to −1.2%/yr; *P* < .001) during 1992 to 2019. The AAI of men (−1.6%/yr; 95% CI: −1.8% to −1.5%/yr; *P* < .001) declined faster than that of women (−1%/yr; 95% CI: −1.2% to −0.8%/yr; *P* < .001). The incidence of men was average 2.16 (range: 1.89–2.53) times more than that of women in the US population.

**Table 4:** Baseline data and univariate analysis of 55,510 GC patients in SEER.

Characteristics	Total (n = 55,510)	Univariate analysis	*P*
Age	67.7 ± 14.03	1.013 (1.013, 1.014)	0
Sex			
Male	34,848 (62.8%)	Reference	
Female	20,662 (37.2%)	0.984 (0.965, 1.003)	.093
Race			
White	37,094 (66.8%)	Reference	
Black	5059 (9.1%)	1.032 (1.000, 1.066)	.051
Asian or Pacific Islander	12,384 (22.3%)	0.746 ((0.729, 0.764)	.000
American Indian/Alaska Native	805 (1.5%)	1.138 (1.055, 1.227)	.001
Unknown	168 (0.3%)	0.460 (0.367, 0.577)	.000
Marital status			
Single	23,047 (41.5%)	Reference	
Married	32,463 (58.5%)	0.818 (0.803, 0.833)	.000
Stage			
Localized	13,260 (23.9%)	Reference	
Regional	19,141 (34.5%)	1.637 (1.594, 1.681)	.000
Distant	18,420 (33.2%)	4.391 (4.273, 4.512)	.000
Unknown	4689 (8.4%)	3.073 (2.961, 3.189)	.000
Grade			
Well (I)	2029 (3.7%)	Reference	
Moderately (II)	11,973 (21.6%)	1.470 (1.390, 1.554)	.000
Poorly (III)	29,696 (53.5%)	1.812 (1.718, 1.912)	.000
Undifferentiated (IV)	1086 (2.0%)	1.836 (1.691, 1.993)	.000
Unknown	10,726 (19.3%)	1.979 (1.870, 2.096)	.000
Primary site			
Non-cardia	39,015 (70.3%)	Reference	
Cardia	16,495 (29.7%)	1.080 (1.059, 1.102)	.000
Surgical therapy			
No	24,952 (45%)	Reference	
Yes	30,558 (55%)	0.321 (0.315, 0.327)	.000
Radiotherapy			
No/unknown	42,348 (76.3%)	Reference	
Yes	13,162 (23.7%)	0.832 (0.814, 0.850)	.000
Chemotherapy			
No/unknown	30,090 (54.2%)	Reference	
Yes	25,420 (45.8%)	0.956 (0.938, 0.974)	.000

GC = gastric cancer, SEER = Surveillance, Epidemiology, and End Results databases.

IBM strikingly increased in the first 2 years for both men and women. Subsequently, IBM started to significantly decrease by 1.4% per year (95% CI: −1.6% to −1.3%/yr; *P* < .001) from 1994, which was consistent with GBD data. IBM of males decreased from 1994 to 2019 (−1.8%/yr; 95% CI: −1.9% to −1.6%/yr; *P* < .001). However, after IBM of females went through a plateau during 1994 to 2000, IBM significantly decreased by 1.5% per year (95% CI: −1.8% to −1.2%/yr; *P* < .001) (Fig. 2B). IBM of men was average 2.30 (range: 2.02–2.69) times more than that of women and the proportion of IBM was significantly higher than that of incidence (*t* = 3.362, *P* = .001). AAPCs of incidence and mortality were shown in Table 5.

**Table 5:** AAPCs of trends in different gender during 1992–2019.

Index	Overall	Male	Female
Incident rates/100,000	−1.3 (−1.4, −1.2)	−1.6 (−1.8, −1.5)	−1 (−1.2, −0.8)
Mortality rates/100,000	1.3 (0.4, 2.2)	1 (0.1, 1.9)	1.4 (0.2, 2.6)
1-yr survival	1.1 (0.9, 1.2)	1.2 (1.0, 1.3)	0.9 (0.7, 1.2)
3-yr survival	1.4 (1.1, 1.7)	1.6 (1.3, 1.9)	1.2 (0.7, 1.6)

AAPCs = average annual percent changes.

### 3.3. Trends of 1-year and 3-year survival in the US

Figure 2C and D show the cancer-specific survival in the US population. The overall 1-YS improved at an annual percent of 1.1% (95% CI: 1%–1.3%/yr), from 42.22% in 1992 to 56.05% in 2018. The 1-YS improved from 42.05% to 57.00% during 1992 to 2018 (1.2%/yr) for males and increased at an annual percent of 0.9% for females. The overall 3-YS improved at an annual percent of 1.4% (95% CI: 1.1%–1.7%/yr), from 22.39% in 1992 to 31.35% in 2018. During the whole period, the 3-YS of males improved from 22.03% to 32.10% (1.6%/yr) and the 3-YS of females increased at an annual percent of 1.2%/yr. All of above *P* values were less than .001. AAPCs of 1-YS and 3-YS were shown in Table 5.

### 3.4. Trends in tumor stage and treatment modality in the US

**Figure 3.**
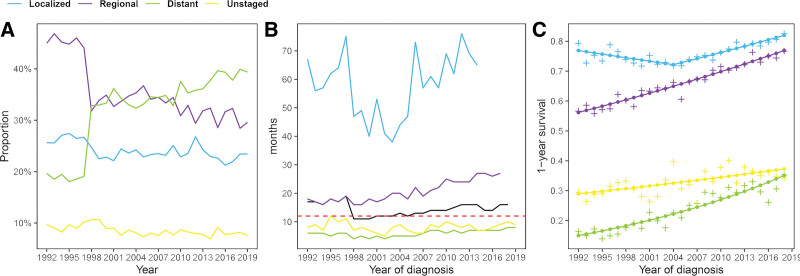
The incidence proportion (A), the median overall survival (B), and 1-yr survival (C) of different stage gastric cancer in the Surveillance, Epidemiology, and End Results 12 program from 1992 to 2019. Black solid line represents overall survival trend of overall patients and red dashed line represents 12 mo. The scattered plus represent the observed value (C). The points and lines were estimated by joinpont model (C).

Details of disease stage are shown in Table 4. The proportion of localized disease was in dynamic balance from 1992 (25.6%; 358/1398) through 2019 (23.5%; 481/2051) (Fig. 3A). The regional disease had a downturn from 1992 (45.0%; 629/1398) through 2019 (29.6%; 607/2051), while distant disease increased from 19.7% (273/1398) to 39.4% (808/2051). The proportion of unstaged disease always maintained small (range: 6.9%–10.7%). The OS substantially decreased in 1997–1998 from 19 to 11 months, subsequently stayed in the plateau and then stably increased (Fig. 3B). Median OS of localized disease could not be assessed after 2015 because of the limited number of end-points. During the study period, the greatest OS improvement was observed in individuals with regional disease, from 18 to 27 months. OS in distant and unstaged disease maintained the low level with the ranges of 6–9 and 5–12 months. The 1-YS curve of localized disease was v-shaped with the APCs decreasing at a rate of 0.5%/yr (95% CI: −0.9% to −0.1%/yr; *P* = .015) during 1992 to 2004 and increasing at a rate of 0.9%/yr (95% CI: 0.6%–1.2%/yr; *P* < .001) during 2004 to 2018. The overall 1-YS increased in regional (1.2%; 95% CI: 1.1%–1.3%/yr; *P* < .001), distant (3.3%; 95% CI: 2.7%–3.9%/yr; *P* < .001) and unstaged disease (1%; 95% CI: 0.5%–1.5%/yr; *P* < .001) in 1992 to 2018 (Fig. 3C).

**Figure 4.**
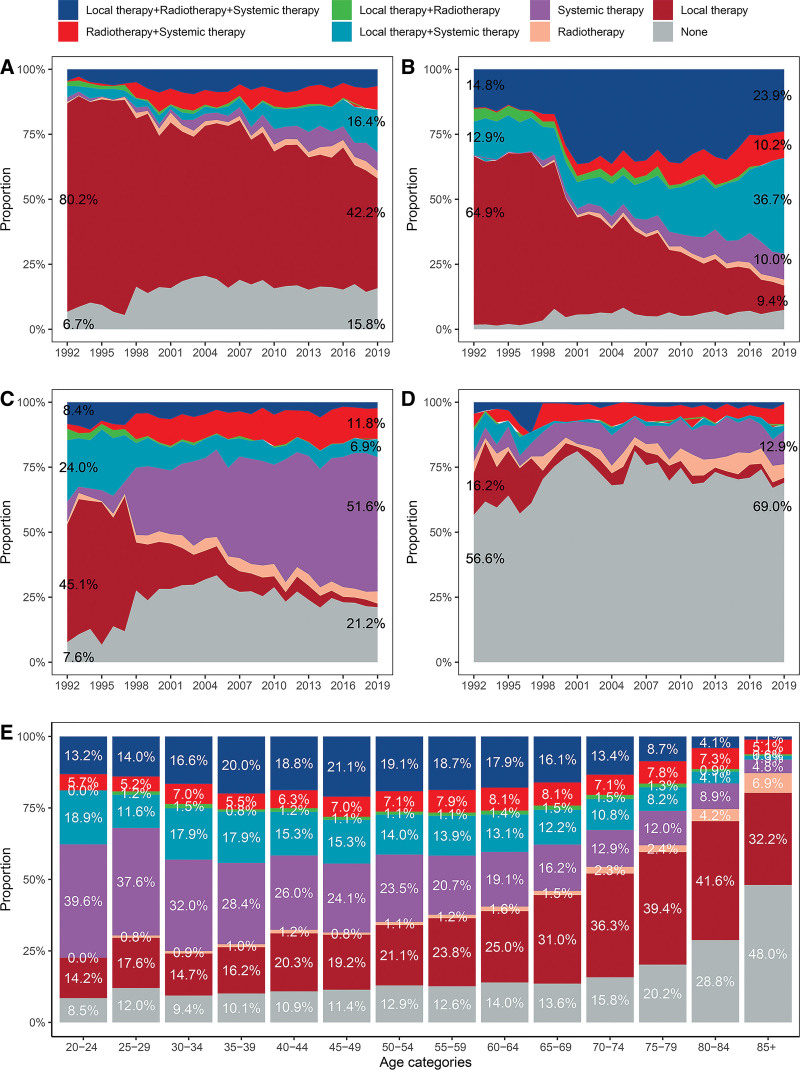
Distribution of treatment modalities by stage and age in the Surveillance, Epidemiology, and End Results 12 program from 1992 to 2019. (A) localized; (B) regional; (C) distant; and (D) unstaged. The percentages of main treatment in 1992 and 2019 are shown in the left and right sides of the picture. (E) Distribution of treatment modalities in each age group. The percentages of every treatment are shown in the picture for better presentation.

The treatment modality distributions by stage during 1992 to 2019 are presented in Figure 4A–D. In total, a significant decrease was found in patients who received single local therapy while a distinct increase was found in patients received systemic therapy, especially for distant disease. The use of combined therapy increased over time in localized and regional disease, especially for local therapy + systemic therapy, radiotherapy + systemic therapy and local therapy + radiotherapy + systemic therapy. Local therapy combined with systemic therapy was gradually replaced by radiotherapy + systemic therapy in distant disease.

Figure 4E shows the treatment modality distributions by age. None of 20 to 24 age group received mere radiotherapy or local therapy + radiotherapy. Administrations of conservative treatment and single local therapy increased with age, whereas mere systemic therapy and local combined with systemic therapy decreased with age. Administrations of mere radiotherapy and radiotherapy combined with either of the two, local or systemic therapy, maintained dynamic stability in a small proportion across all age groups. The combination of radiotherapy, local and systemic therapy slightly increased from 20–24 age group through 45–49 age group and then decreased with age, visibly for older than 70.

## 4. Discussion

GC still constitutes a major global health burden, especially in China and so on.^[6]^ GC ranked second in cancer burden for both incidence and mortality in China, while it correspondingly ranked the fourteenth and the fifteenth in the US in 2019.^[2]^ This study firstly described and compared the concrete trends of GC incidence, IBM, and DALYs in the US and China. The age-period-cohort models were adopted with GBD data, the world’s most comprehensive data of surveys, censuses, vital statistics, and other health-related data^[7]^ annually updating from 1990 through 2019 to elaborate period and cohort effects of trends. The cases from SEER 12 registries (1992–2019) were used to verify the trends, support the survival estimates and manifest the use of multiple management among specific groupings.

The incidence, mortality and DALYs of China were continuously 4 to 6 times higher than those of the US during the observational period due to considerable regional variation. The three indicators of the US have decreased since 1990. The indicator declines of Americans younger than 40 were slight and even with fluctuations. The incidence of Chinese 20 to 35 showed a general uptrend while IBM and DALYs have apparently fallen in 20th century. Increasing use of screening programs has contributed to this phenomenon to a certain extent. As young patients were more respond to genetic carcinogens,^[8]^ early-onset GC was considered as a good model to study genetic alterations related to the carcinogenesis process. The incidence, mortality and DALYs of Chinese elder than 35 showed fluctuation with a slow decline, a rapid increase and then a downtrend. Bao et al^[9]^ estimated the general decline with a slight rise (1995–2000) of incident and death rates in urban Shanghai for both males and females during 1973 to 2010, which was partially overlapped with this study. The fluctuation may be associated with the increase in the medical visit rate promoted by the basic medical insurance implementation and gradual cover since 1998. The GC mortality decreased faster in Chinese rural areas than the urban areas.^[10]^ This study confirmed the significance of healthy lifestyle development, medical technology improvement, endoscopic screening routine, and relevant gastric diseases treatments in economically undeveloped areas. A Korean study of more than 20 million individuals received the endoscopy, verified that sex, age group, hospital type, metropolitan area, and history of gastric diseases including atrophic gastritis, ulcer, intestinal metaplasia, gastric polyp, and other gastric diseases were significantly associated with GC detection.^[11]^ Each contributing factor is modifiable and context-specific. Where a medical condition is homogeneous, lifestyle or hereditary factors may be a crucial role, and inversely where subjective factors are consistent, the level of medical care is the deciding factor.

The period and cohort effects of incidence (Fig. 1G and H) were affected by behavioral, environmental, occupational and metabolic risk factors. On the basis, the period and cohort effects of mortality (Fig. 1I and J) were also affected by treatment. The RRs of the period and cohort effect of incidence in the US, and mortality in the US and China continued to decrease over the whole period. Both period and cohort effects of incidence fluctuated in China. This may be associated with better sanitation has been offset by other factors including overwork, obesity or food sharing.^[12,13]^ Horizontally, the RRs of cohort effects for incidence and mortality in the US went down faster than China before 1950, but the opposite was observed since 1950. China broke from its feudal past in 1949, established a new political structure, boosted the economy progress and modernized the country. Socioeconomic status has been proved to be strongly associated with GC incidence and mortality,^[13]^ and the decrease can be related to behavioral, occupational, metabolic risk factors and treatment modality in a rapid-changing socioeconomic environment.^[9]^ Vertically, the constantly decreasing RRs of cohort effect may also due to the better education that the later birth cohorts received than the earlier cohorts, and therefore had a stronger awareness of health and disease prevention.^[14]^ We discuss strategies to describe international gaps and promote prevention efforts, which have broader public health implications.

GC burden derived from old patients is a notable public problem. The GC incidence and mortality in both countries as well as DALYs for Chinese younger than 70 and Americans increased with age. There were such age-dependent increases resulting from the population aging and average lifespan improvement. It was obvious that DALYs of Chinese older than 70 decreased with age from 1990 to 2004. In fact, it is still necessary to make more efforts on the elderly prognosis, especially for 70 to 84 Chinese.

Heavier GC burden on male patients was observed than that on female patients. GC ranked second in male-specific DALY rankings and fifth in female rankings.^[2]^ Both age adjusted rate and IBM of men were higher than those of women in this study, especially for IBM, which was similar to Colquhoun et al.^[15]^ Previous studies proposed that high GC incidence in males resulted from heavy smoking, high-sodium diet, occupational exposure and drug-use habits.^[1]^ However, this study found that 1- and 3-YS of men in the US increased more strikingly than those of women. The GC intervention strategies need to be tailored to specific subgroups to maximize the potential beneficial effect.^[16]^

The recommendations for disease stages have undergone many changes, leading to unstable trends in certain stage disease. The regional disease had a visible fall from 1997 to 1998, and meanwhile, distant disease had a substantial increase. The fifth edition AJCC staging for GC was published in 1997 and incorporated a major revision with respect to nodal staging.^[17]^ After that, the proportion of GC respectively in localized, regional, distant stages or unknown stage remained relatively stable during 1998 to 2010 as Thrift and El-Serag.^[4]^ Subsequently, regional disease decreased with fluctuation while distant disease increased with fluctuation. The distant-stage disease accounted for quite a bit (39.92%) of GC diagnosis in the US in 2019. It was remarkable that 39% of patients with metastatic disease was found at diagnosis in a Dutch study.^[18]^

The prognosis for GC patients was strongly related to the stage at diagnosis (Fig. 2E). Early detection can improve survival. Considering the actual situation that most patients have already undergone regional or distant metastases at diagnosis, only through high risk group screening can we realize early detection and provide patients with the chance of radical resection. The endoscopic biopsy has always been the golden standard for early diagnosis, acting as the secondary prevention strategy.^[8]^ Updated endoscopic technology makes contributions^[19]^ but risk factors such as the COVID-19 pandemic may delay the diagnosis to deteriorate the profile and decelerate survival improvement finally. Thus, it is of high significance for high-risk patients across the countries with heterogeneous incidence to timely take gastroscopy.

Considering the variable distribution of treatment by age or stage, the optimal options for specific groups should be studied. The prospective randomized trials of surgical resection of GC suggested poorer survival in older patients with limited functional reserved and high risk of complications.^[20]^ Administration of local therapy dramatically increased with age in this study. In addition, palliative or no treatment was more likely to be seen in the elderly. The uses of combined therapy and mere chemotherapy were generally less in old patients than in young patients.

Administration of single local therapy went down significantly in each stage disease. Prognosis remained poor with 5-year overall survival rates of 33% to 50% because patients with stage I to III GC underwent the local therapy alone.^[21]^ Administration of systemic therapy was increasing, such as single modality or it combined with radiotherapy in each stage, or it combined with local therapy just in local and regional stage. Satisfyingly, systemic therapy had promising data when used in the perioperative setting, for example, standard FLOT (5-fluorouracil, leucovorin, oxaliplatin, and docetaxel) chemotherapy regimen in combination with targeted therapy (Trastuzumab) had a 3-year OS rate of 82.1%^[22]^ for patients with locally advanced disease. The untreated, unresectable, non-HER2-positive gastric, gastro-esophageal junction, or oesopahgeal adenocarcinoma treated with immunotherapy (Nivolumab) combined with chemotherapy (oxaliplatin and capecitabine every 3 weeks or leucovorin, fluorouracil, and oxallipatin every 2 weeks) had OS of 13.1 months. And chemotherapy alone had OS of 11.1 months.^[23]^ A meta-analysis identified perioperative radiochemotherapy had no superior OS improvement in comparison to perioperative chemotherapy (HR = 0.93; 95% CI: 0.82–1.06; *P* = .28)^[24]^ in terms of locally advanced disease. The trial found postoperative chemoradiotherapy can improve OS (36 month vs 27 months; *P* = .005) and progression-free survival (30 months vs 19 months; *P* < .001) compared with single local therapy for patients with resectable GC.^[25]^ To our knowledge, the combination of the three therapies is increasing in localized and regional diseases, underlining the importance of multidisciplinary assessment and treatment in determining the most optimal treatment strategy for GC patients. Given the poor postoperative treatment compliance, it is not difficult to speculate that neo-adjuvant treatment may be the focus of future studies.^[26]^ Above all, treatment patterns of different stage diseases have gone through big changes over time and guided the direction of further research.

There were some limitations to this study. First, the specific stage, treatment distribution and survival in China were not described. A precise interpretation would be provided if comprehensive data were included. Secondly, there were some inevitable data misses in the SEER database. Unknown or palliative therapy accounted for a large proportion of Americans elder than 85. The generalizability of this conclusion is still subjected to be tested. Thirdly, major causes, such as *Helicobacter pylori* infection were not described in the database. The relationship between GC prevalence and risk factor management still needs to be further illustrated. Last, considering the limited data or insignificance in univariate analysis of multiple factors, such as race, tumor location and grade, we could not perform stratified analyses of these factors.

## 5. Conclusion

The progress is imperative in the war against GC. According to epidemiological characteristics, early- and later-onset GC (younger than 40 or older than 70) draw more attention because of large room for OS improvement. It is necessary to strengthen the timely screening of specific populations, such as male and economically undeveloped area. The multidisciplinary team participation and increasing combined strategy are crucial to control disease progression of specific subgroups. The constructed theory can predict the progress with good performance, which is meaningful to individualized management and might help inform cancer control planning.

## Author contributions

LZ, HD and LZ conceived and designed the study. YZ and NL collected the data. XZ and XK analyzed and interpreted the data. LZ wrote the manuscript. LZ and PZ revised the manuscript.

**Conceptualization:** Yunhui Zhou, Xinyi Zhou, Lin Zhou.

**Data curation:** Ningning Liu.

**Formal analysis:** Linlin Zheng, Hang Ding, Lin Zhou.

**Validation:** Xiaohua Kong.

**Writing – original draft:** Linlin Zheng.

**Writing – review & editing:** Ping Zhao.
